# Mathematical Modeling of Sub-Cellular Asymmetry of Fat-Dachsous Heterodimer for Generation of Planar Cell Polarity

**DOI:** 10.1371/journal.pone.0097641

**Published:** 2014-05-19

**Authors:** Mohit Kumar Jolly, Mohd Suhail Rizvi, Amit Kumar, Pradip Sinha

**Affiliations:** Department of Biological Sciences and Bioengineering, Indian Institute of Technology Kanpur, Kanpur, India; Tel Aviv University, Israel

## Abstract

Planar Cell Polarity (PCP) is an evolutionarily conserved characteristic of animal tissues marked by coordinated polarization of cells or structures in the plane of a tissue. In insect wing epithelium, for instance, PCP is characterized by *en masse* orientation of hairs orthogonal to its apical-basal axis and pointing along the proximal-distal axis of the organ. Directional cue for PCP has been proposed to be generated by complex sets of interactions amongst three proteins - Fat (Ft), Dachsous (Ds) and Four-jointed (Fj). Ft and Ds are two atypical cadherins, which are phosphorylated by Fj, a Golgi kinase. Ft and Ds from adjacent cells bind heterophilically via their tandem cadherin repeats, and their binding affinities are regulated by Fj. Further, in the wing epithelium, sub-cellular levels of Ft-Ds heterodimers are seen to be elevated at the distal edges of individual cells, prefiguring their PCP. Mechanisms generating this sub-cellular asymmetry of Ft-Ds heterodimer in proximal and distal edges of cells, however, have not been resolved yet. Using a mathematical modeling approach, here we provide a framework for generation of this sub-cellular asymmetry of Ft-Ds heterodimer. First, we explain how the known interactions within Ft-Ds-Fj system translate into sub-cellular asymmetry of Ft-Ds heterodimer. Second, we show that this asymmetric localization of Ft-Ds heterodimer is lost when tissue-level gradient of Fj is flattened, or when phosphorylation of Ft by Fj is abolished, but not when tissue-level gradient of Ds is flattened or when phosphorylation of Ds is abrogated. Finally, we show that distal enrichment of Ds also amplifies Ft-Ds asymmetry. These observations reveal that gradient of Fj expression, phosphorylation of Ft by Fj and sub-cellular distal accumulation of Ds are three critical elements required for generating sub-cellular asymmetry of Ft-Ds heterodimer. Our model integrates the known experimental data and presents testable predictions for future studies.

## Introduction

**Figure 1:**
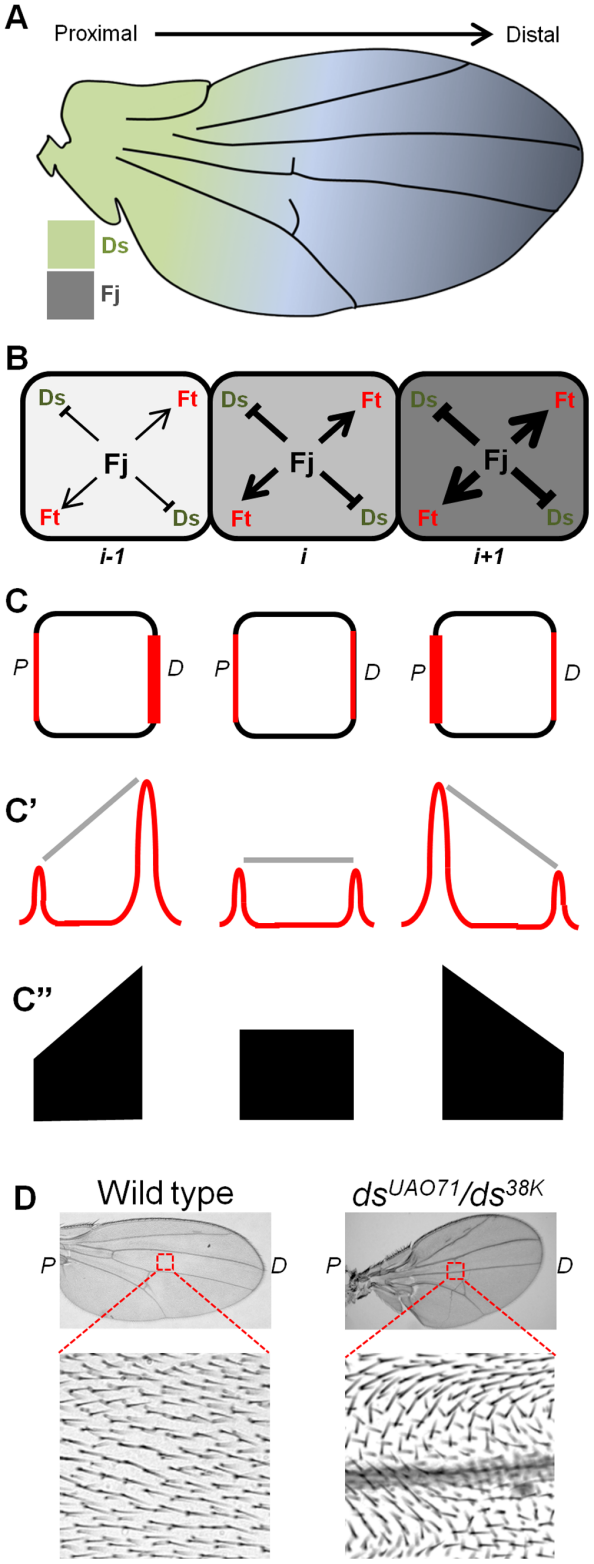
Fat (Ft)-Dachsous (Ds)-Four-jointed (Fj) system regulates PCP in *Drosophila* wing. (A) Schematic of *Drosophila* wing showing opposing expression gradients of Ds (green) and Fj (grey) while expression of Ft (not shown) remains uniform. Arrow marks the proximal-distal (P/D) axis of the wing. (B) Arrangement of cells (boxes) seen in the contexts of proximo distally increasing gradient of Fj expression (grey) as well its activity; the latter represented by arrows and hammers symbolizing its activating and repressive roles, respectively, while thicknesses of these lines indicate relative strengths of these activities. (C) Different concentrations of the Ft-Ds heterodimer (represented by thickness of red line) at the proximal and distal edges of the cells provide distinct intensity plot. (C′) A line joining the peaks of these two levels of Ft-Ds heterodimer at the proximal and distal edges would be proximally or distally inclined depending on the concentrations of the Ft-Ds heterodimer at these edges. This line would, however, be horizontally placed when concentrations of the Ft-Ds heterodimer are equal at the proximal and distal edges. (C′′) Simplified visual representations of the concentrations of Ft-Ds heterodimer at the proximal and distal edges of the epithelial cells seen in (C′). (D) Orientation of wing hairs in wild type and in dachsous (*ds*) mutant adult wings.

Planar Cell Polarity (PCP) represents coordinated orientation of cells or structures in an axis within the tissue plane. Most animal organs display PCP, which when perturbed lead to developmental anomalies such as open neural tubes, polycystic kidneys, and even can cause deafness due to loss of planar polarization of the cilia in the inner ear [1]. PCP has been extensively studied in *Drosophila* wing, eye, and in the adult abdomen [2]. *Drosophila* wing, in particular, is an ideal model system to study PCP, which is mirrored by projection of actin-rich trichomes (hairs) from the distal edges of its individual cells (Figure 1D) [3], [4].

Three evolutionarily conserved proteins, namely, Fat (Ft), Dachsous (Ds) and Four-jointed (Fj), the so called Ft-Ds-Fj system or global regulators of PCP, generate a tissue-level directional cue thereby linking the axis of polarization of individual cells with that of the tissue/organ: for instance, projection of the wing hairs along the proximal-distal axis in *Drosophila* wing [5]. A second class of proteins consisting of Frizzled (Fz), Flamingo (Fmi), Van Gogh (Vang), Prickled (Pk) and Dishevelled (Dsh), referred to as core PCP proteins, propagate PCP signals from cell-to-cell [2], [6]. Finally, several down-stream effectors, including cytoskeletal proteins, respond to these signals from global regulators and core proteins to establish PCP [7]. Upon loss of global signal, as in *ft, ds, fj* mutants, PCP could be seen to be coordinated amongst neighboring cells; however, the direction of their polarity is often not aligned with the tissue/organ axis thereby giving rise to swirling patterns (Figure 1D)[8]–[10]. Loss of activities of the core proteins, in contrast, abolish PCP altogether in individual cells; for instance, wing hairs in *fz* mutant may grow out of the centers of the cells, instead of their distal edges [6].

Ft, Ds and Fj display intricate interactions with each other. Ft and Ds, for instance, are large atypical cadherins that bind heterophilically between adjacent cells via their tandem cadherin repeats [5]. Fj, a golgi kinase, phosphorylates both Ft and Ds [11], [12], which in turn increases affinity of Ft for Ds and decreases that of Ds for Ft [13], [14]. Also, expression levels of Ds and Fj vary across the tissue; in the wing primordium, for instance, these form opposing gradients where Fj expression is higher in its distal than that of its proximal domain, and vice-versa for Ds (Figure 1A) [10], [15], [16]. These opposing gradients of Fj and Ds expressions have been seen to be critical for establishing PCP [9], [17]. Further, Ft-Ds heterodimer also displays a sub-cellular distal enrichment, thereby, resulting in its asymmetric localization (polarization) in the proximal and distal membrane of the cells preceding their overt display of PCP through asymmetric localization of the core proteins such as Fz [18], [19]. Ft-Ds heterodimer asymmetry thus could be the earliest and coarse signals for cell polarization that are eventually strengthened by sub-cellular asymmetry of the core PCP proteins and their signaling. A critical question that has not been answered yet is how the known interactions within these three elements of the Ft-Ds-Fj system translate into a sub-cellular asymmetry of the Ft-Ds heterodimer.

A mathematical modeling approach can offer insights into how a set of biochemical interactions at a local (sub-cellular) scale could impact a global pattern such as the coordinated PCP in an entire organ like the insect wing. Several mathematical models have been developed to understand various aspects of PCP [20]. The first integrated modeling and experimental study in PCP [21] offered a model for intercellular feedback regulations amongst core proteins to explain ‘domineering effect’ [22] – a phenomenon wherein perturbation in PCP in a group of cells lacking (mutant), for instance, Fz protein activity was relayed to wild type neighboring cells. This study proposed that domineering effect can simply emerge from the dynamics of the known intercellular and intracellular interactions among the core proteins (Fz, Vang, Pk and Dsh). This model further suggested that a directional cue provided by Ft-Ds-Fj system is crucial for explaining the observed PCP patterns [21]; however, it did not elucidate how the Ft-Ds-Fj system as such generates this cue. Further, attempts at mathematical modeling of PCP captured the mechanism of action of the core proteins, and strengthened the idea that a weak tissue-level directional cue is important for obtaining the experimentally observed PCP patterns[23]–[30]. A recent phenomenological model proposes how Ft-Ds heterodimer formation in a group of cells in the tissue can induce collective polarization over the entire tissue; however, this model did not factor the crucial question of the role played by tissue-level expression gradients of Ds and Fj for the regulation of PCP [31]. Since none of these models factored all activities of the members of Ft-Ds-Fj system, our current understanding of their contribution in PCP regulation remains far from complete.

Here, using a mathematical modeling approach, we have simulated the experimentally identified interactions amongst the members of Ft-Ds-Fj system to reveal their impact on sub-cellular asymmetry of Ft-Ds heterodimer. We have developed an Ordinary Differential Equation (ODE)-based model by taking into consideration the known interactions in Ft-Ds-Fj system. These elements are: (a) Ft-Ds heterophilic binding, (b) opposing tissue-level gradients of Ds and Fj and, finally, (c) phosphorylation of Ft and Ds by Fj kinase. We show that Fj gradient, phosphorylation of Ft by Fj, and, sub-cellular distal accumulations of Ds are three essentials for establishing sub-cellular asymmetry of Ft-Ds heterodimer in individual cells.

## Results and Discussion

### Model Formulation

We assumed that cells in *Drosophila* wing are arranged along rows that are oriented in a single proximal-to-distal axis. We modeled one such row of cells for deciphering the sub-cellular asymmetry of Ft-Ds heterodimer (Figure 1B). For the ease of visual representation, we have denoted asymmetry in sub-cellular enrichment of Ft-Ds heterodimer in proximal and distal edges of individual cells by trapezoid shapes where the slope, either distally or proximally inclined, mirrors its enrichment at the distal or proximal cell membrane, respectively. However, when Ft-Ds heterodimers are equally enriched in proximal and distal edges, their symmetric distribution is represented in a rectangular shape (Figure 1C, C′ and C′′). We have assumed the physical gradient of Fj to mean a gradient for its kinase activity; thus, in our simulations, the Fj kinase activity gradient is synonymous with its actual spatial gradient of expression (Figure 1A). Further, due to a distally enriched graded expression of Fj in *Drosophila* wing, its kinase activity too is shown to be graded along the proximal-distal axis of the wing (Figure 1B).

Since Ds and Fj are present in opposing gradients in the wing, total concentration of Ds (represented by 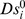 for *i^th^* cell) decreases from proximal to distal end of the wing. On the other hand, total concentration of Ft (represented by 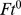
_)_ is taken to be same in all cells. We assume that during establishment of PCP, total concentrations of Ft and Ds (sum of both phosphorylated forms of Ft and Ds) remain uniform in each cell. However, due to the gradient of Fj kinase activity, levels of phosphorylated Ft and Ds turn out to be different for each cell. Thus, the only variables considered during establishment of PCP are phosphorylated and unphosphorylated states of Ft and Ds. Fj phosphorylates both Ds and Ft and their rates of phosphorylation are designated 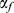 and 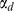, respectively, while their dephosphorylation rates are designated as 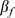 and 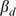, respectively. The effect of Fj on phosphorylation of Ft and Ds in *i^th^* cell (Figure 1B) is considered to follow a monotonically increasing Hill’s function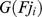, which is given by
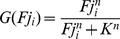
(1)where 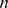 and 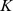 are two model parameters.

Since Fj is expressed in a tissue-level gradient, its kinase activity is considered to increase linearly as we move along the proximal-distal axis of the wing. Hill’s function thus ensures that effect of Fj on phosphorylation of Ft and Ds is higher in the distal end as compared to that in the proximal. This difference in the strength of regulation is shown by the increasing thickness of arrows and hammers in Figure 1B as we move distally along the row of cells. Therefore, we can write
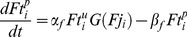
(2)




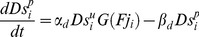
(3)where 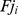,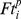,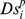, 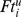 and 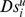 represent the concentrations of Fj, phosphorylated Ft, phosphorylated Ds, unphosphorylated Ft, and unphosphorylated Ds, respectively in the *i* ^th^ cell.

**Figure 2:**
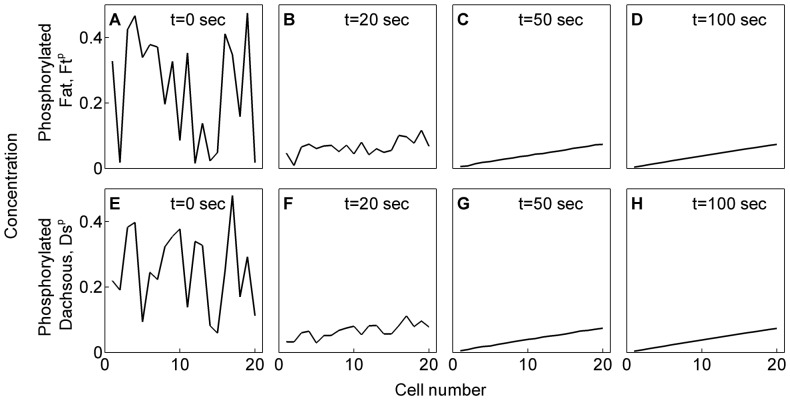
Evolution of phosphorylated Ft (*Ft^p^*) and phosphorylated Ds (*Ds^p^*). (A–D) Evolution of sub-cellular concentrations of phosphorylated Ft (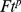) and that of (E–H) phosphorylated Ds (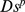) in cells over time. (A) and (E) show the randomized initial concentrations of 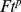 and 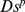, respectively.

We performed the analysis with randomly initialized values of concentrations of phosphorylated Ft and Ds (Figure 2A–H). In the SI (File S1), we show that the levels of phosphorylated Ft (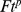) and Ds (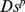) attain stable steady state values over time irrespective of the initial values of 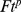 and 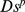. Thus, our results hold good for any set of initial values of 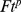 and 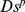 in the row of cells considered.

In our analysis, we normalized the concentrations of phosphorylated and unphosphorylated Ft by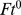 (the total concentration of Ft in each cell). Also, we normalized the levels of Ds and Fj with their respective total concentrations in the most distal cell of the row. We can calculate the concentration of unphosphorylated Ds in the *i^th^* cell as
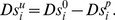
(4)


Since phosphorylated Ds (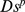) has decreased affinity to bind to Ft, while phosphorylated Ft (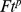) has increased affinity to bind to Ds [13], [14], we assumed that the interaction between phosphorylated Ft and unphosphorylated Ds (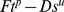) is much greater than those between other forms of Ds and Ft (i.e. 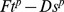, 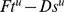 and 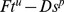). Thus, 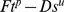 is the most abundant form of Ft-Ds heterodimer. Therefore, hereafter, by Ft-Ds heterodimer, we refer to the binding of phosphorylated Ft,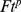, of one cell with unphosphorylated Ds, 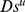, of its adjacent cell. Thus, two different binding events contribute to the formation of Ft-Ds heterodimer at the edge between two given cells A and B – (a) 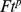 of A binds with 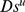 of B, and (b) vice-versa i.e., 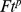 of B binds with 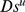 of A. Concentration of this Ft-Ds heterodimer is referred to as *C*. If the rates of formation and dissociation of the heterodimers are 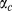 and 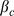
_,_ respectively, then the rates of heterodimer formation at the two edges of the *i^th^* cell are given by equations (5) and (6)
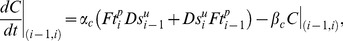
(5)

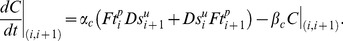
(6)


Equation (5) represents the heterodimer formation at the proximal edge of *i^th^* cell, or the edge shared between *i^th^* cell and *(i−1)^th^* cell, whereas Equation (6) represents the heterodimer formation at the distal edge of *i^th^* cell, or the edge shared between *i^th^* cell and *(i+1)^th^* cell. Therefore, the rate of evolution of sub-cellular asymmetry in the heterodimer formation between the two edges of the *i* ^th^ cell is given by equation (7).
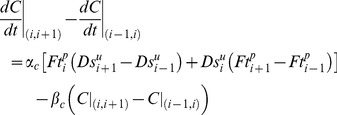
(7)


Furthermore, we assumed that the heterodimer formation does not influence the levels of available Ft and Ds monomers (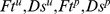) in the cell. Also, we did not consider any feedback on monomer concentration due to heterodimer formation, since heterodimer formation and phosphorylation of unphosphorylated monomers (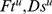) happens in different cellular compartments – the former at cell edges, and the latter at the Golgi by the Fj kinase.

### Parameter Values and Initial Conditions

Representative model parameter values for the simulations are listed in Table 1. The sensitivity analysis (see Figures S1, S2 in File S1) showed that the conclusions drawn from the model do not change for a large range of parameter values.

**Table 1:** List of parameters used in the theoretical model.

N	20
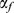	2
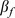	0.1
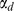	2
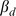	0.1
N	1
K	250
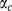	1
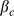	0.1

### Ft-Ds-Fj Interactions Culminate in Sub-cellular Asymmetric Enrichment of Ft-Ds Heterodimer at the Distal Edges

The model developed was numerically simulated for 20 cells. Simulations for phosphorylated Ft,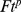 (Figure 2 A–D), and phosphorylated Ds,
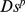
(Figure 2 E–H), show that their levels in a cell achieve a steady state over time. The expression for time evolution of 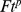 and 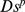 is given in SI (File S1). We also simulated the model for many different sets of parameters, and found quite similar results for the set of parameters as present in Table 1. Variations in the parameters, namely, n, K, 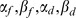
displayed only a modest effect on our results for a very large range (Figures S1, S2 in File S1). We also varied the relative strength of the kinase activity of Fj for its substrates, Ft and Ds, by changing the parameters of the two Hill’s functions in equations (2) and (3), and found our results not to be overtly sensitive to these changes too (Figure S2).

**Figure 3:**
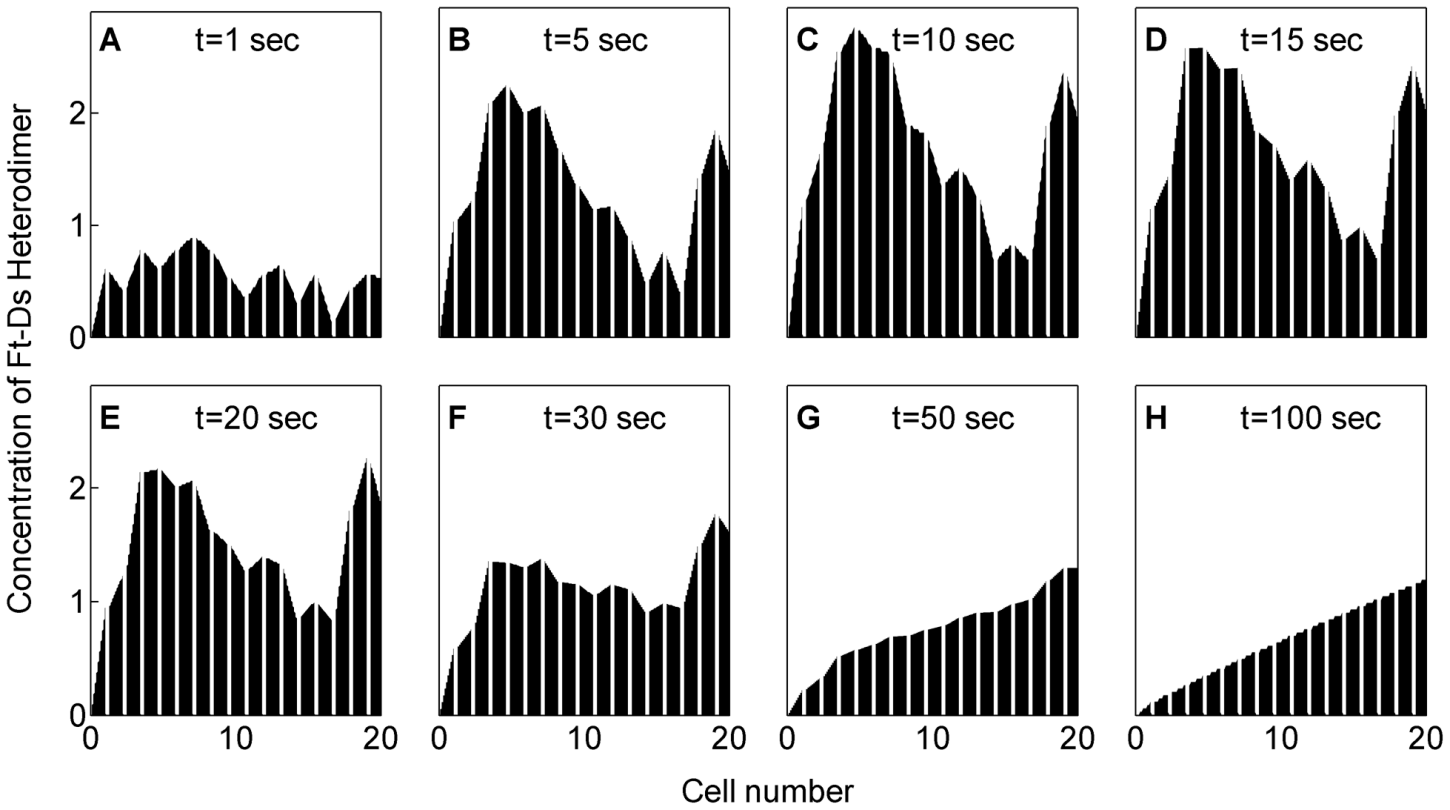
Evolution of asymmetric localization of the Ft-Ds heterodimer in cells over time. (A–H) Evolution of the asymmetric levels of Ft-Ds heterodimer in cells. (A) shows the initial uncoordinated levels of Ft-Ds heterodimer while that shown in (H) represent the final steady state.

Concentration of Ft-Ds heterodimer (or the 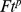
−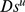 heterodimer) across the tissue evolved with time, starting with random uncoordinated values to a coordinated steady state at the cell edges (Figure 3 A–H). In all panels of Figure 3, each trapezoidal column represents levels of Ft-Ds heterodimer on the two edges of a single cell, where the apparent slope of the trapezoid, as explained in Figure 1 C, C’ and C′′, mirrors disparity in the concentrations of Ft-Ds heterodimer at proximal and distal cell edges, revealing their asymmetric sub-cellular enrichments. Thus, in a steady state, concentration of Ft-Ds heterodimer for each cell in wild type scenario at its distal edge is more than that on its proximal edge (Figure 3H). Also, since distal edge of a cell abuts the proximal edge of its distal neighbor, value of Ft-Ds heterodimer concentration at the distal edge for one cell equals that of the proximal edge of its neighbor (Figure 3H).

These simulations thus show that interactions within the Ft-Ds-Fj system culminate in a sub-cellular distal enrichment of Ft-Ds heterodimer (Figure 3H). This is corroborated by recent experimental observations in the wing that display sub-cellular asymmetric localizations of the Ft-Ds heterodimer [18], [19]. In these studies, Ft and Ds were seen to localize asymmetrically on the two edges of the cell, with low Ft and high Ds on the distal edge, and high Ft and low Ds on the proximal [18], [19].

The model presented here appears to be robust in generating consistent polarity patterns over a large range of parameter values, and variations in initial conditions of randomized Ft and Ds activity (see SI File S1). Besides a sub-cellular graded pattern of Ft-Ds heterodimer, the model also predicts a distally elevated endogenous tissue-level graded pattern of Ft-Ds heterodimer formation (Figure 3H), which has not yet been observed experimentally. This spatial gradient in Ft-Ds heterodimer concentration is likely to have domain-specific impact on PCP of the organ. Uniformly high levels of Ft or Ds, on the other hand, would alter this graded pattern of Ft-Ds heterodimer, which in turn could preferentially affect PCP in the proximal, but not the distal wing – endogenous concentration Ft-Ds heterodimer being low in the former. Previous experimental results appear to corroborate this fallout of our simulation. Thus, when Ft or Ds was overexpressed in their respective naïve contexts: that is, in *ft* or *ds* mutant background, PCP in the distal but not in proximal domain was rescued [16], [32].

Also, since the Ft-Ds heterodimer represents phosphorylated Ft (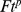) bound with unphosphorylated Ds (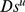) (see Model formulation), a sub-cellular asymmetry (distal enrichment) of the heterodimer would also mean distally enhanced phosphorylated Ft (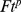). It may be noted that previous speculation [9], [16] about asymmetric Ft activity (which is synonymous with its phosphorylated state) has not been validated yet. Immunochemical detection using antibody that discriminates between phosphorylated and unphosphorylated Ft could provide an assay for distally enhanced (asymmetric) Ft activity.

### Fj Gradient, but not Ds Gradient, Plays an Essential Role in Attaining Sub-cellular Asymmetry of Ft-Ds Heterodimer

**Figure 4:**
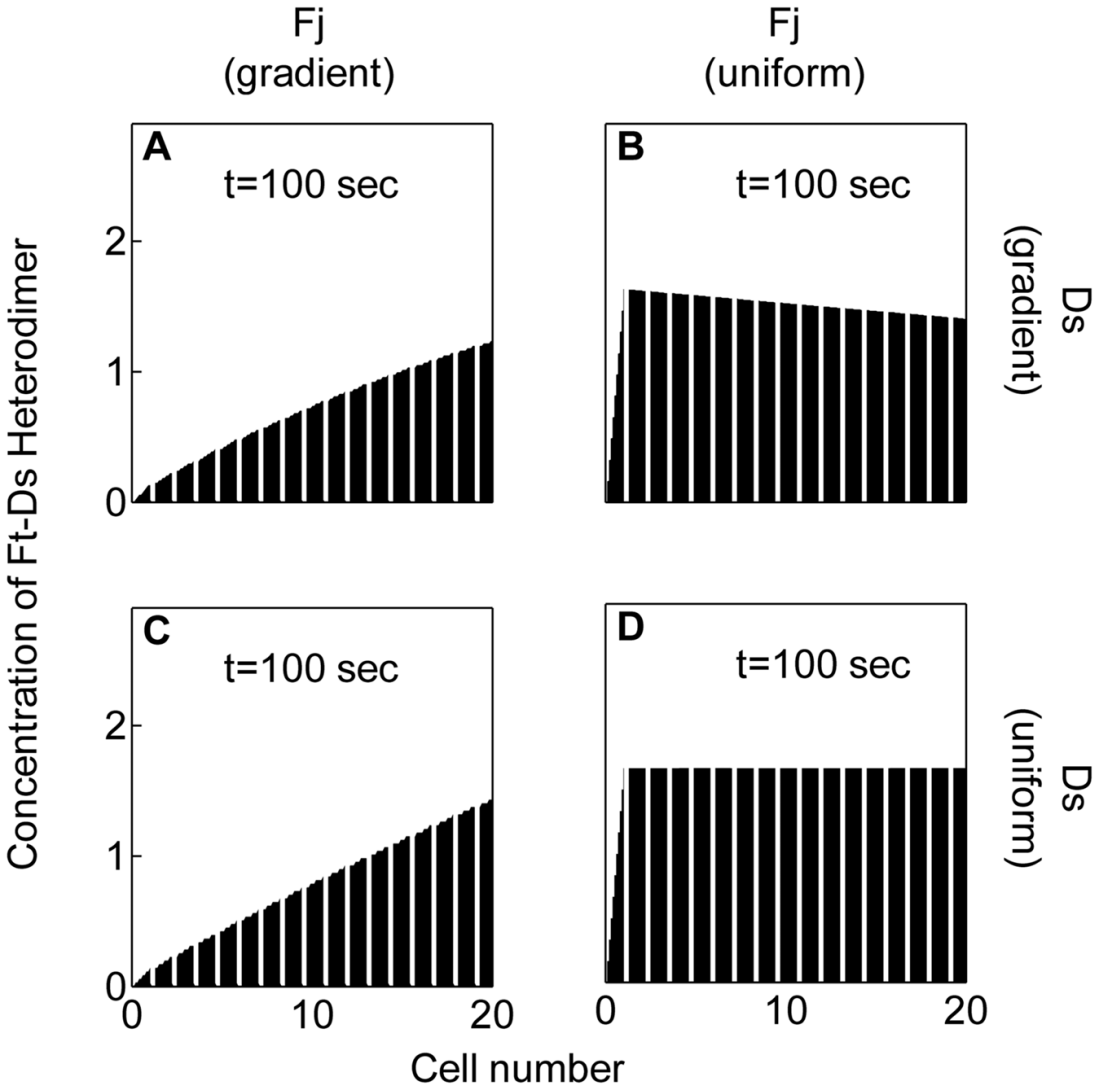
Fj gradient, but not that of Ds, is essential for generating the asymmetric localization of the Ft-Ds heterodimer. (A) Asymmetric localization of Ft-Ds heterodimer (distally heightened top of trapezoid) is seen when both- Fj and Ds are expressed in gradient. Data shown here is the same as that shown in Figure 3H. (B) Uniform over-expression of Fj results in a loss of asymmetry of Ft-Ds heterodimer (flattened top) in all cells, although Ds expression is maintained in a gradient. (C) Conversely, uniform overexpression of Ds does not affect Ft-Ds heterodimer asymmetry when Fj expression remains in a gradient. (D) When both Fj and Ds are uniformly overexpressed, asymmetry of Ft-Ds heterodimer is lost.

To test if the distally elevated gradient of Fj at the tissue level in the developing wing [11], [16] is critical for attaining sub-cellular asymmetric localization of the Ft-Ds heterodimer, we examined the consequences of flattening Fj gradient *in silico.* Indeed, when tissue-level expression gradient (or activity gradient; since we assume the expression and kinase gradients of Fj to be synonymous) of Fj was abolished by its uniform overexpression, it led to a loss of sub-cellular asymmetry in Ft-Ds heterodimer localization (Figure 4B). We expect this loss of sub-cellular asymmetry of Ft-Ds heterodimer to cause the loss of sub-cellular asymmetry of developing wing cells. Our findings corroborated well with the experimental observations wherein overexpression of Fj (leveled Fj gradient) in the wing leads to the loss of membrane localization of the an unconventional myosin Dachs (D) [33]. It may be noted that D is a downstream component of the Ft-Ds-Fj system, which co-localizes with Ds on the distal edges of the cells [33], and thus presents a readout of sub-cellular asymmetry and cell polarization [18], [19], [34].

Like Fj, Ds too is expressed in a tissue-level gradient, but with a proximal elevation [10], [16]. We examined the consequences of flattening this Ds gradient by its overexpression, and found that a flattened Ds gradient does not disturb sub-cellular asymmetry of Ft-Ds heterodimer, as long as the Fj gradient is intact (Figure 4C). This would imply that when Fj displays tissue-level gradient, expression of Ds alone – irrespective of its expression being uniform or in gradient at a tissue level – suffice for PCP. We then predicted that uniform overexpression of Ds would rescue polarity defects, for instance, in *ds* mutant wings since their endogenous Fj gradient remains unaffected; this was indeed observed experimentally [16]. On the other hand, a flattened Fj gradient disturbs sub-cellular asymmetry of the heterodimer, even when Ds gradient is intact (Figure 4B). Further, when both Ds and Fj gradients are flattened, the pattern observed (Figure 4D) appears akin to that caused by flattening of Fj (Figure 4 B) but not the one presented by flattened Ds gradient (Figure 4C). Therefore, Fj gradient, but not that of Ds, is essential for attaining sub-cellular asymmetry of Ft-Ds heterodimer. In other words, Ds gradient by itself (without the Fj gradient) is not enough to give correct patterning. Together, these findings suggest that while Ds is required for generation of Ft-Ds asymmetry and cell polarization, its tissue-level gradient *per se* may not be critical for PCP.

### Phosphorylation of Ft by Fj is Essential in Achieving Sub-cellular Asymmetry of Ft-Ds

**Figure 5:**
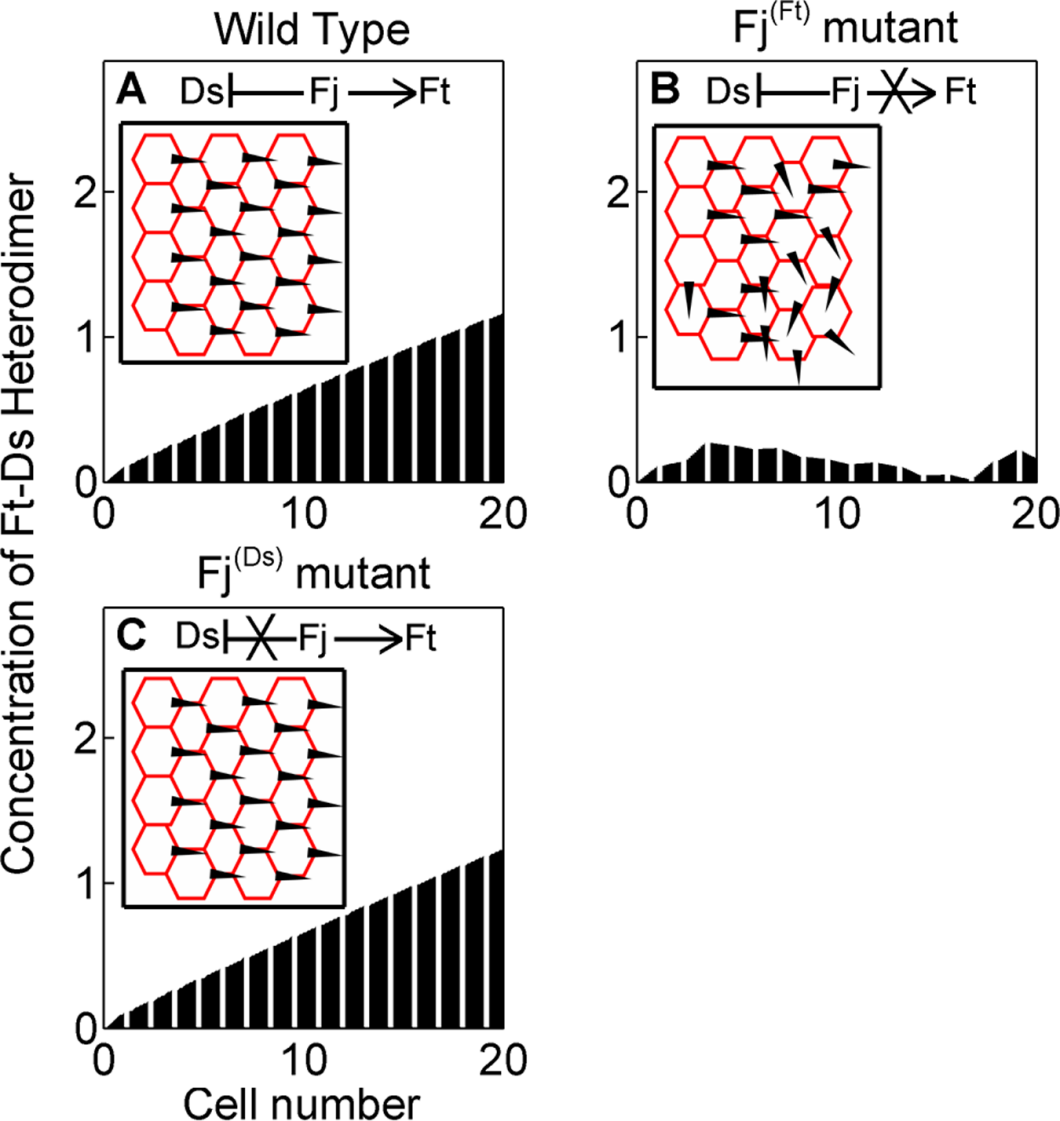
Phosphorylation of Ft by Fj kinase is necessary for Ft-Ds asymmetry. (A) Wild type Fj activity displays characteristic distal enrichment of Ft-Ds heterodimers. Data shown here is same as that shown in Figure 3H. Inset displays the ordered projection of *Drosophila* wing hair in proximal-distal axis. Arrow and hammer indicate the activating and repressing roles of kinase Fj against Ft and Ds, respectively. (B) Asymmetry of Ft-Ds heterodimer is disrupted when phosphorylation of Ft by Fj kinase is lost in Fj^(Ft)^ mutant. This would result in swirling of wing hairs (inset). (C) Loss of kinase activity of Fj against Ds in Fj^(Ds)^ mutant, however, does not influence asymmetric localization of Ft-Ds heterodimer. Orientations of wing hairs now remain intact (inset) like that of wild type wing (A).

Fj kinase phosphorylates both Ft and Ds and regulates binding of Ft to Ds in two ways – by inhibiting binding of Ds with Ft on one hand and by promoting binding of Ft with Ds on the other (Figure 5A) [13], [14]. This opposing action of Fj on Ds and Ft has been proposed to establish sub-cellular polarity with high fidelity [6], [14]. We asked whether the phosphorylation of both Ft and Ds by Fj kinase is necessary for producing sub-cellular Ft-Ds asymmetry. *In silico* mutation, which retained Fj kinase activity (phosphorylation) against Ds but lacked that against Ft (Fj^(Ft)^ mutant) disrupted PCP across the tissue (Figure 5B). The converse, meaning intact Fj kinase activity against Ft but loss of that against Ds (Fj^(Ds)^ mutant), however, did not disrupt PCP (Figure 5C). Thus, while phosphorylation of Ft by Fj kinase appears to be an essential for achieving Ft-Ds sub-cellular asymmetry, redundancy of phosphorylation of Ds by Fj as seen in our simulation may confer robustness to polarization process across the tissue.

### Sub-cellular Polarity of Unphosphorylated Ds (*Ds^u^*) Amplifies Sub-cellular Asymmetry of Ft-Ds Heterodimer

Distal edge of a cell abuts proximal edge of its distal neighbor or, in other words, the edge shared between *i^th^* cell and *(i+1)^th^* cell is distal for *i^th^* cell but proximal for *(i+1)* ^th^ cell. At this edge, Ft-Ds heterodimer can be formed in two different orientations–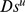 (unphosphorylated Ds) of the *i^th^* cell binds with 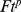 (phosphorylated Ft) of the *(i+1)^th^* cell (denoted by 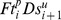), or
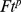
of the *i^th^* cell binds to 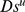
of *(i+1)^th^* cell (denoted by 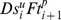), as shown in Equation (6). Similarly, heterodimers formed at proximal edge of the *i^th^* cell, or the edge shared between *i^th^* and *(i−1)^th^* cell, can be formed in two possible orientations - 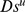
of the *i^th^* cell binds with 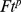 of the *(i−1)^th^* cell (denoted by 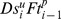) or 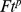 of the *i^th^* cell binds to 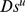 of the *(i−1)^th^* cell (denoted by 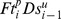), as shown in Equation (5).

We calculate the difference in heterodimer concentration between distal and proximal edges of the *i^th^* cell at steady state, denoted by 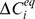 and given by the equation given below (For details, see equation (S4) in File S1)-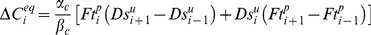
(8)


The term 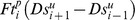 represents the difference between the distal and proximal sub-cellular concentrations of the heterodimer formed by 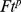
of the *i^th^* cell (with 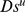 of (*i+1)^th^* cell and with 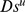 of (*i−1)^th^* cell, respectively). Therefore, the term 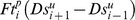 denotes sub-cellular polarity of 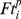 or phosphorylated Ft in the *i^th^* cell. By similar reasoning, the other term 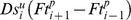, denotes sub-cellular polarity of 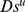
in *i^th^* cell. Hence, it is evident that overall sub-cellular asymmetry of heterodimer in *i^th^* cell, 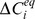, are contributed by sub-cellular polarity of phosphorylated Ft, 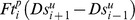, and that of unphosphorylated Ds, 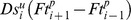.

While sub-cellular asymmetry of the Ft-Ds heterodimer has been shown experimentally [18], [19], previous experiments did not resolve if sub-cellular polarity of 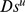 and that of 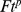
contribute equally to sub-cellular asymmetry of the heterodimer. We thus used *in silico* mutations to investigate this crucial question. In all simulations till now, we have considered contributions of these two mechanisms – sub-cellular polarity of 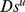 and that of 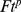
– to be implicitly equal. However, in order to investigate whether they contribute equally in generating the sub-cellular asymmetry of Ft-Ds heterodimer, we introduced a weight factor 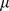
in the equation as
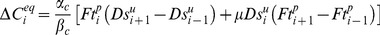
(9)


**Figure 6:**
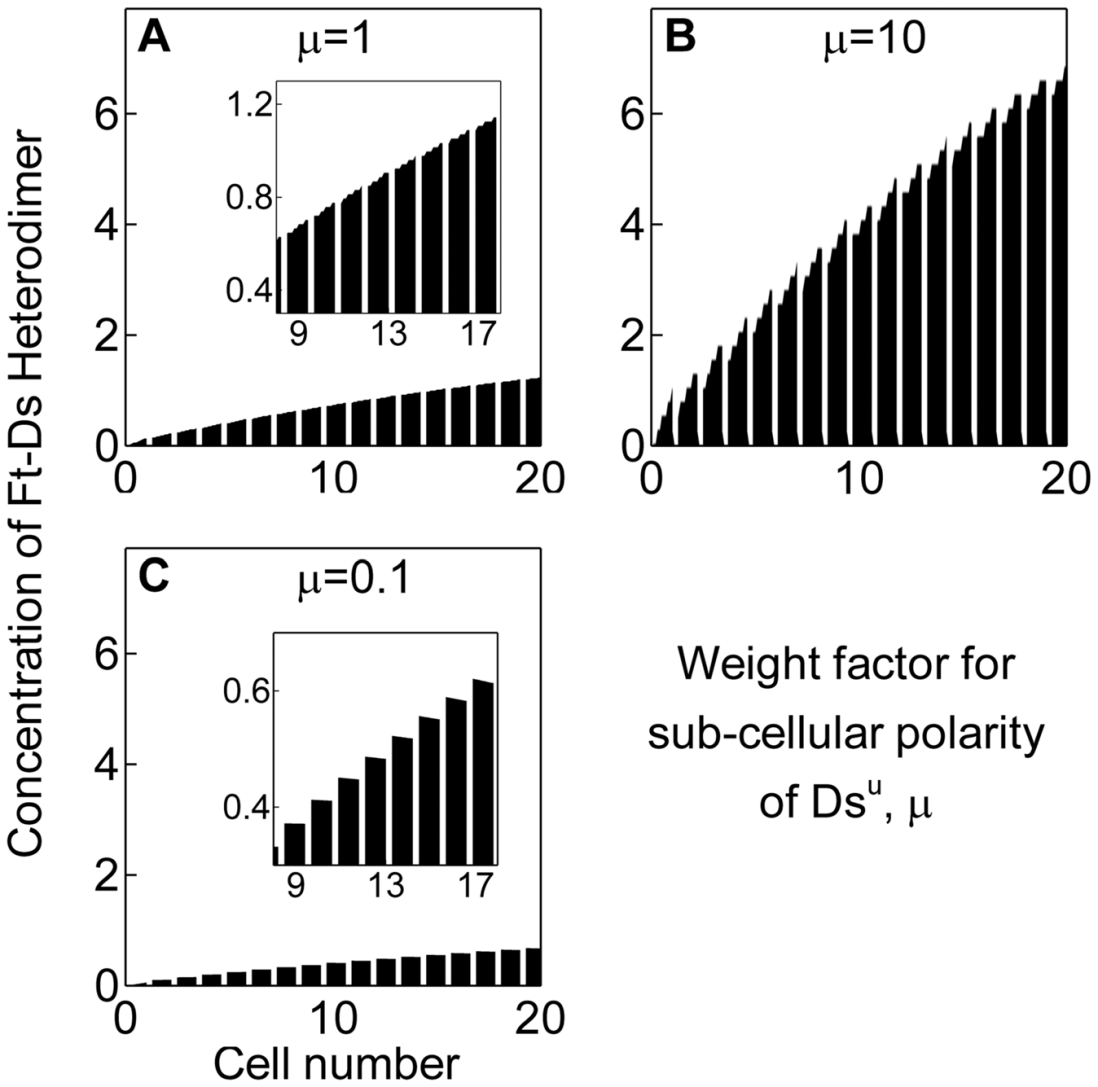
Sub-cellular asymmetry of 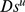
enriches asymmetry of Ft-Ds heterodimer. (A) Equal contributions of sub-cellular asymmetries of 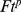 and 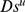 result in asymmetric localization of Ft-Ds heterodimer (µ = 1). Inset shows the enlarged view with inclined tops of the trapezoids. Data shown here is same as that shown in Figure 3H. (B) Higher contribution of sub-cellular asymmetry of 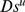 augments the asymmetric localization of Ft-Ds heterodimer (steeper top of the trapezoids) in cells (µ = 10). (C) Higher contribution of 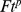 sub-cellular asymmetry, however, results in diminished or even reversed asymmetry of Ft-Ds heterodimer in cells (µ = 0.1). Inset shows the enlarged view with flatter tops of the trapezoids indicating weak asymmetry of Ft-Ds heterodimer.

When contribution of sub-cellular polarity of 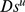 in determining overall sub-cellular polarity of the cell was taken to be much higher (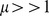 in the *in silico* mutations) than that of 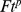, we found sub-cellular asymmetry of heterodimers to be much more pronounced (Figure 6B) as compared to a condition when their contributions were considered to be equal – that is 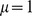 (Figure 6A). This suggests that sub-cellular polarity of 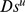 amplifies sub-cellular asymmetry of Ft-Ds heterodimer. On the contrary, when contribution of sub-cellular polarity of Ft was taken to be much higher, or that of sub-cellular polarity of 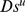 was taken to be much smaller (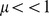), asymmetric localization of heterodimer was lost, or even reversed in some distal most cells (Figure 6C). This reinforces the interpretation that sub-cellular polarity or asymmetric localization of 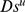 largely contributes to sub-cellular distal enrichment of the Ft-Ds heterodimer.

As explained above, Ft-Ds heterodimer at the distal edge of *i* ^th^ cell can be formed in two alternative orientations. However, it has not been resolved experimentally which of these two orientations (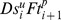 or 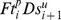) is the preferred orientation of the Ft-Ds heterodimers at the all cell edges within a tissue. It is likely that these two alternative orientations of the heterodimer are linked to sub-cellular polarity of phosphorylated Ft and also that of unphosphorylated Ds. As mentioned above, sub-cellular polarity of unphosphorylated Ds, or 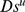 in a cell is given by 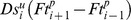. Similarly, sub-cellular polarity of 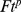
in a cell is given by 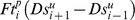. We found that overall sub-cellular asymmetry of the Ft-Ds heterodimer is amplified by sub-cellular polarity of unphosphorylated Ds (Figure 6B); therefore, the preferred orientation of the heterodimer at all cell edges in the tissue would be the one that corresponds to sub-cellular polarity of unphosphorylated Ds, i.e. 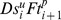 and not to that of phosphorylated Ft, i.e. 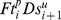. Thus, our results indicate that heterodimer formed at the distal edge of a cell is between its unphosphorylated Ds and phosphorylated Ft of its distal neighbor (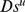 of *i^th^*cell with 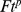 of *(i+1)^th^* cell, or 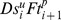). This presents an interesting possibility, namely, that within a cell, unphosphorylated Ds is asymmetrically localized towards its distal edge, while phosphorylated Ft is localized towards its proximal edge. Immunochemical detection using an antibody that discriminates between phosphorylated and unphosphorylated forms of Ft and Ds can help validate this prediction.

In a recent phenomenological model of PCP [31], subcellular asymmetry of Ft-Ds heterodimer and a consequent tissue–scale collective cell polarization were achieved without considering the Fj gradient. Rather, Ft-Ds heterodimer asymmetry at the cell edges was obtained by assuming mutual inhibition between the two alternative orientations of the heterodimers while promoting their respective orientations. In other words, 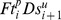 promotes formation of more of its kind while inhibiting formation of 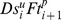; likewise, 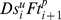 does the converse [31]. While this remains an attractive possibility, available experimental data do not offer support for such mutual inhibitory and self-promoting roles of Ft-Ds heterodimers. In contrast, our model does not assume such a mutually inhibitory and self-promoting role of Ft-Ds heterodimers and yet achieves their sub-cellular asymmetry at the cell edges by factoring Fj gradient (Figure 4A, C). A resolution of the question as to which one of these two models approximates an actual cellular event would demand experimental investigation. It may be further noted that this model proposed that all cell edges in a tissue would prefer any one out of two orientations of the heterodimers (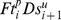 and 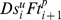) without a specific preference of one over the other [31]. Given that Fj kinase modulates the affinities of Ds and Ft for each other, a consideration of Fj gradient made in our model shows that 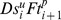, and not 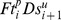, is the preferred orientation of Ft-Ds heterodimer at the cell edges (Figure 6B, C).

### Caveats and Concluding Remarks

A caveat of our model is its unidimensionality: interactions within the Ft-Ds-Fj system have been captured in only one dimension within the plane of tissue. Adopting this model in two dimensions presents challenges in terms of defining the direction of polarity. Further, our analysis explains sub-cellular asymmetry of Ft-Ds heterodimer; deciphering interactions of the global regulators (Ft-Ds-Fj system) with the core proteins (Fz, Vang, Pk, Dsh) remains outside the scope of our model. These caveats notwithstanding, our analysis shows that a mathematical model of PCP at an appropriate level of abstraction can provide novel and non-intuitive insights about the dynamics of the system [35] and testable predictions. For instance, our simulations predict a tissue-level spatial gradient of the Ft-Ds heterodimer concentration (Figure 3H). We also predict that sub-cellular polarity of Ds amplifies sub-cellular asymmetry of Ft-Ds heterodimer (Figure 6B, 6C), thereby revealing a fundamental feature of a planar polarized cell. Similarly, we predict that phosphorylation of Ft by Fj is essential for establishing PCP, and suggest specific mutational studies to validate the same (Figure 5B, 5C).

In conclusion, our mathematical model reveals that a set of known interactions within the Ft-Ds-Fj system, namely, gradient of Fj expression, phosphorylation of Ft by Fj kinase, and sub-cellular Ds localization generate an asymmetric enrichment of the Ft-Ds heterodimer in distal cell membrane. This could in turn provide PCP cue across the tissue. Our results are in agreement with previous experimental findings which reveal that Fj gradient, but not Ds gradient, is required for establishing PCP [16], [33], and that PCP is more resistant to overexpression of Ds or Ft in the distal region of the wing as compared to the proximal [16], [32]. It may be further noted that global regulators and core proteins display tissue-specific interactions. In *Drosophila* wing and eye, for instance, global regulators acts upstream of the core module [6] – the latter in turn act on tissue-specific effectors for PCP. In the abdomen, on the other hand, Ft-Ds-Fj system is proposed to signal directly to the tissue-specific PCP effectors [36], ‘bypassing’ the core proteins. It is likely that the role of Ft-Ds-Fj system modeled here holds true for PCP regulation in both these scenarios: namely, its down-stream signaling directly via tissue-specific PCP effectors [6], or indirectly through the core regulators [36].

Future iterative experimental and computational efforts in multi-scale modeling of PCP by linking the models of the Ft-Ds-Fj system with those of the core proteins can help resolve their individual contributions in establishing PCP, and gain comprehensive insights about the regulation of this evolutionarily conserved phenomenon of coordinated cell orientation [20].

## Methods

We discretized the difference-differential equations using forward difference scheme in the time domain. Time evolution of 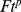,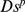 and Ft-Ds heterodimer concentrations were obtained from the discretized equations using MATLAB (Mathworks, USA). For random initialization of 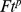 and 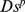
_,_ uniformly distributed random numbers were used using MATLAB’s in-built random number generator.

## Supporting Information

File S1
**Supplementary Information file that shows the expression for** 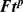 **and 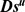 as a function of time, includes SI** **figures 1** **and** **2** **, and analyzes the robustness of the mathematical model.**
(DOCX)
