# Supplementary material for: Mathematical Modeling of Sub-Cellular Asymmetry of Fat-Dachsous Heterodimer for Generation of Planar Cell Polarity
Source: PLoS One. 2014 May 19;9(5):e97641. doi: 10.1371/journal.pone.0097641 (PMC4026444; doi:10.1371/journal.pone.0097641)
Supplement: File S1 — Supplementary Information file that shows the expression for and as a function of time, includes SI figures 1 and 2 , and analyzes the robustness of the mathematical model. (DOCX) [file pone.0097641.s001.docx]

**Mathematical Modeling of Sub-cellular Asymmetry of Fat-Dachsous Heterodimer for Generation of Planar Cell Polarity**

Mohit Kumar Jolly^1,2,*,#^, Mohd. Suhail Rizvi^1,*^, Amit Kumar^1^ and Pradip Sinha^1,#^

^1^Department of Biological Sciences and Bioengineering, Indian Institute of Technology Kanpur, Kanpur 208016, India

^2^Present address: Center for Theoretical Biological Physics and Department of Bioengineering, Rice University, Houston TX 77005, USA

**Supplementary Information**

In order to study the influence of the values of the model parameters and initial conditions of the system we solved the equations (2) and (3) of the main text. From equations (2) and (3) we get

This shows that as the time progresses the concentrations of the phosphorylated Ft and Ds in a cell reach a stable state which is determined by the Fj concentration in that cell as well as the reaction coefficients $\alpha_{f}$, $\beta_{f}$, $\alpha_{d}$ and $\beta_{d}$(Figure S1). Further, this also demonstrates that the conclusions of the study are not dependent on the initial conditions of $Ft^{p}$and $Ds^{p}.$

Now from equation (5) the equilibrium concentration of Ft-Ds heterodimer at the proximal edge of the *i^th^* cell is given by

$$\left.C^{eq}\right|_{\left(i-1,i\right)}=\frac{\alpha_{c}}{\beta_{c}}\left(Ft_{i}^{p}Ds_{i-1}^{u}+Ds_{i}^{u}Ft_{i-1}^{p}\right)$$

From here we get the equilibrium difference in the heterodimer concentrations at the two edges of the *i^th^* cell as

$$\Delta C_{i}^{eq}=\frac{\alpha_{c}}{\beta_{c}}\left[Ft_{i}^{p}\left(Ds_{i+1}^{u}-Ds_{i-1}^{u}\right)+Ds_{i}^{u}\left(Ft_{i+1}^{p}-Ft_{i-1}^{p}\right)\right]$$

This shows that the asymmetry in the Ft-Ds heterodimer concentration does not depend on rate constant of heterodimer formation and degradation. However, the distal end of each cell will have higher concentration of Ft-Ds heterodimer in comparison to that on proximal end only if

$$Ds_{i}^{u}\left(Ft_{i+1}^{p}-Ft_{i-1}^{p}\right)>Ft_{i}^{p}\left(Ds_{i+1}^{p}-Ds_{i-1}^{p}\right)$$

For simplicity, we have assumed the tissue level gradient of total Ds to be shallow in the above relation. Substituting the expressions for $Ft_{i}^{p}$ and $Ds_{i}^{p}$ from equations and into inequality gives that the formation of the Ft-Ds heterodimer on the two edges of *i^th^* cell will be asymmetrical if following condition is satisfied

$$\frac{1}{\lambda G\left(Fj_{i}\right)+1}\frac{1}{\left(\eta G\left(Fj_{i+1}\right)+1\right)\left(G\left(Fj_{i-1}\right)+\frac{1}{\eta}\right)}>\frac{G\left(Fj_{i}\right)}{G\left(Fj_{i}\right)+\frac{1}{\eta}}\frac{1}{\left(\lambda G\left(Fj_{i+1}\right)+1\right)\left(G\left(Fj_{i-1}\right)+\frac{1}{\lambda}\right)}$$

where $\lambda=\frac{\alpha_{d}}{\beta_{d}}$ and $\eta=\frac{\alpha_{f}}{\beta_{f}}$. Figure S1 shows the permissible values for $\lambda$and $\eta$for the asymmetric formation of Ft-Ds heterodimer. For small values of the *Fj* the asymmetric heterodimer formation is observed for a large range of parameter values (Figure S1) which indicates a high robustness of the system.

The small values of λ and η correspond to the absence of the regulation of the phosphorylation of *Ds* and *Ft* by *Fj* respectively. In case of very small η *i.e.* the case when *Fj* regulates the phosphorylation of *Ds* only, we need to have the following for the asymmetric localization of Ft-Ds heterodimer on the cell boundaries

$$\left(G\left(Fj_{i+1}\right)G\left(Fj_{i-1}\right)-G\left(Fj_{i}\right)^{2}\right)\lambda^{2}+\left(G\left(Fj_{i+1}\right)+G\left(Fj_{i-1}\right)-G\left(Fj_{i}\right)\right)\lambda+1>0$$

which may not always hold.

On the other hand, for very small λ *i.e.* the case when *Fj* regulates the phosphorylation of *Ft* only, we need to have

$$\frac{1}{\left(\eta G\left(Fj_{i+1}\right)+1\right)\left(G\left(Fj_{i-1}\right)+\frac{1}{\eta}\right)}>0$$

which is always true. Conditions and demonstrate that the dual regulation of the *Fj* is not critical for the establishment of polarity. Moreover, the regulation of phosphorylation of *Ft* by *Fj* is more crucial than that of *Ds* by *Fj.*

As the expression of *Fj* is in a gradient manner is the wing, our conclusions hold as long as function $G$ is monotonically increasing function (see equation ). Therefore changing the parameter *n* of the Hill’s function does not change the findings of the study. However, for large values of *n* it takes system longer to reach the equilibrium state (Figure S2).

Also, by changing the values of other parameters from those mentioned in Table 1, the steady state behavior of the system remains the same, thus demonstrating the model to be very robust (Figure S2). Neither did changing the initial conditions for solving the equations (2) and (3), i.e. the initial values of phosphorylated Ft and Ds have any effect on steady state behavior of the system.


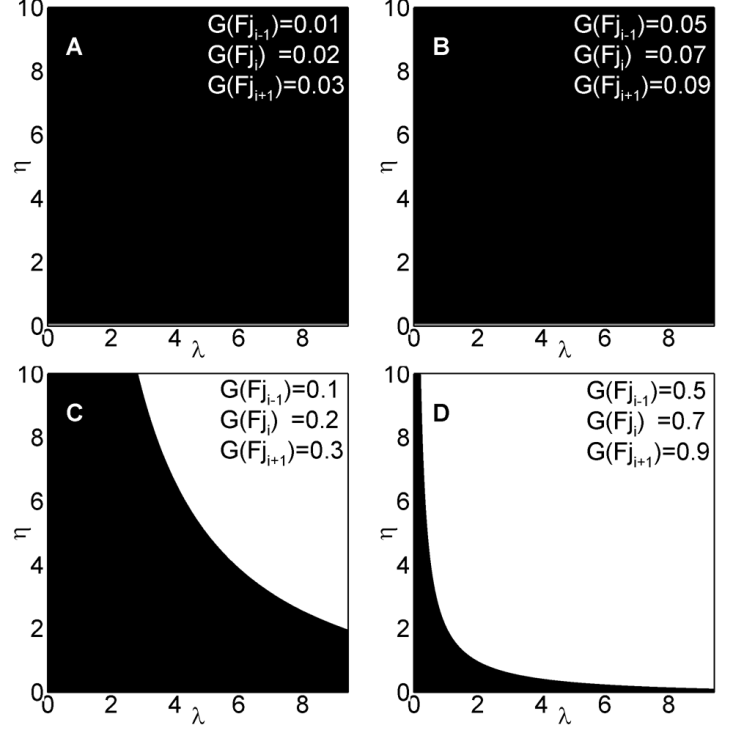


**Figure S1-** **Values of phosphorylation and dephosphorylation rates of Ft and Ds for asymmetric formation of Ft-Ds heterodimer.** (A-D) Ratios of phosphorylation and dephosphorylation rates of Ft and Ds, defined as $\lambda=\frac{\alpha_{d}}{\beta_{d}}$ and $\eta=\frac{\alpha_{f}}{\beta_{f}}$, respectively, which lead to the asymmetric formation of Ft-Ds heterodimer (shown in black). In case of low concentrations of Fj, the asymmetry in Ft-Ds heterodimer is not affected by the values of rates of phosphorylation and dephosphorylation of Ft and Ds (A and B). However, when the levels of Fj are very high, the range of values of $\lambda$ and $\eta$ for which asymmetry in the Ft-Ds heterodimer is established, gets smaller (C and D).

*
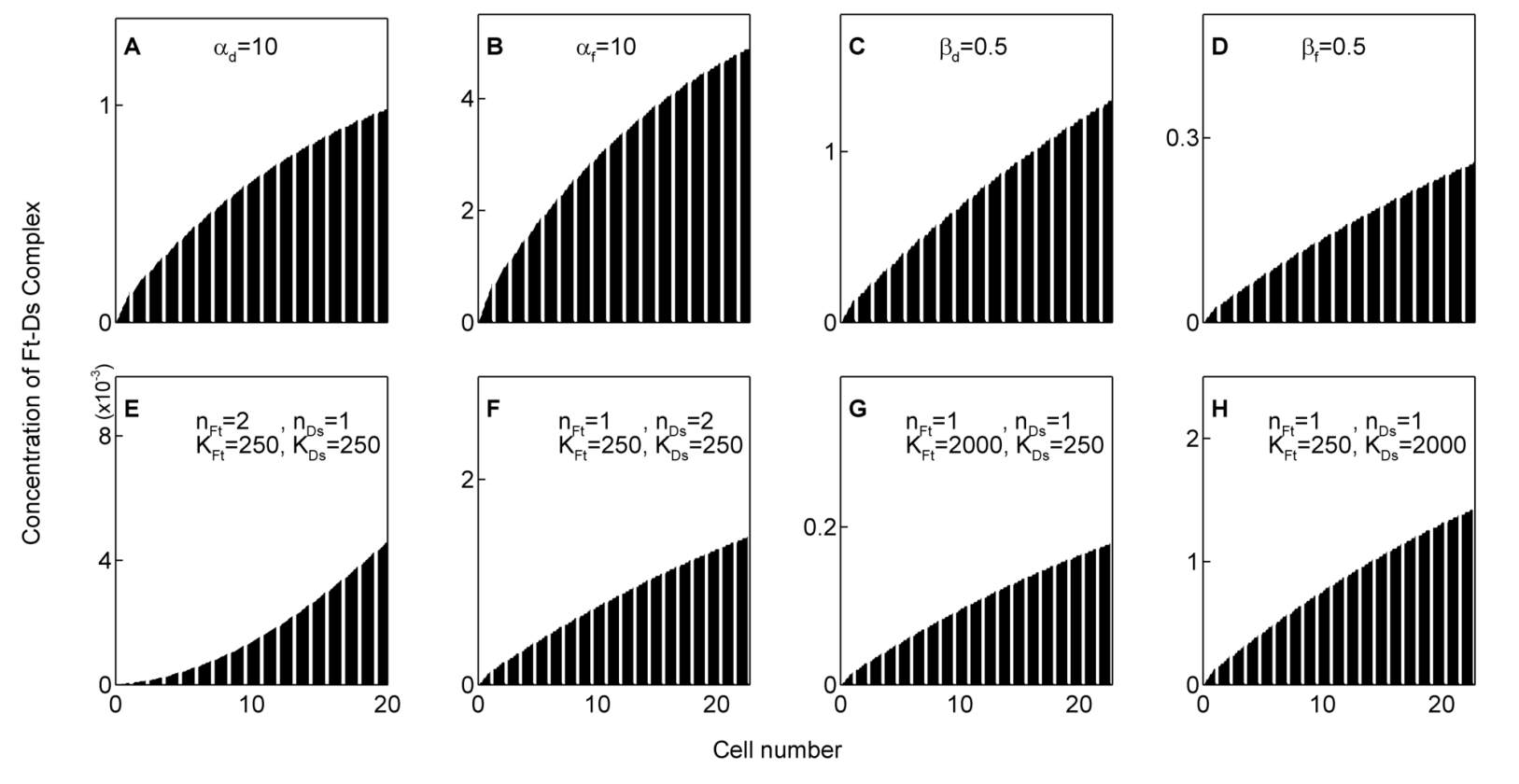
*

**Figure S2- Asymmetric Ft-Ds heterodimer formation is independent of model parameters.** (A and B) Increase in the values of the phosphorylation rates of Ds and Ft by Fj, denoted by $\alpha_{d}$(A) and $\alpha_{f}$(B), respectively, does not result in loss of asymmetry of Ft-Ds heterodimer (inclined tops of the trapezoids). (C and D) Increase in the dephosphorylation rates of the Ds and Ft, denoted by $\beta_{d}$(C) and $\beta_{f}$(D), respectively, also maintains the asymmetric enrichment of Ft-Ds heterodimer. (E-H) Changing the parameters of Hill’s function also results in asymmetric Ft-Ds heterodimer formation. In these panels, the subscripts represent the unequal Hill’s function parameters corresponding to Ft and Ds, which indicate the strength of kinase activity of Fj towards Ft and Ds. However, note that in all these cases, although the asymmetry in the Ft-Ds heterodimer is preserved, the pattern of asymmetry does not remain same.
